# SC3s: efficient scaling of single cell consensus clustering to millions of cells

**DOI:** 10.1186/s12859-022-05085-z

**Published:** 2022-12-12

**Authors:** Fu Xiang Quah, Martin Hemberg

**Affiliations:** 1 Wellcome Sanger Institute, Wellcome Genome Campus, Hinxton, CB10 1SA UK; 2 The Gurdon Institute, University of Cambridge, Tennis Court Road, Cambridge, CB2 1QN UK; 3 Present Address: Evergrande Center for Immunologic Diseases, Harvard Medical School and Brigham and Women’s Hospital, 75 Francis Street, Boston, MA 02115 USA

**Keywords:** k-Means clustering, Streaming clustering, scRNAseq

## Abstract

**Background:**

Today it is possible to profile the transcriptome of individual cells, and a key step in the analysis of these datasets is unsupervised clustering. For very large datasets, efficient algorithms are required to ensure that analyses can be conducted with reasonable time and memory requirements.

**Results:**

Here, we present a highly efficient k-means based approach, and we demonstrate that it scales favorably with the number of cells with regards to time and memory.

**Conclusions:**

We have demonstrated that our streaming k-means clustering algorithm gives state-of-the-art performance while resource requirements scale favorably for up to 2 million cells.

**Supplementary Information:**

The online version contains supplementary material available at 10.1186/s12859-022-05085-z.

## Background

Technological advances have paved the way for single cell RNAseq (scRNAseq) datasets containing several million cells [1]. Such large datasets require highly efficient algorithms to enable analyses at reasonable times and hardware requirements [2]. A crucial step in single cell workflows is unsupervised clustering, which aims to delineate putative cell types or cell states based on transcriptional similarity [3]. The most popular methods for unsupervised clustering of scRNAseq data are the Louvain and Leiden algorithms. They represent cells as a neighborhood graph where densely connected modules are identified as clusters [4]. However, these methods can be biased by a poorly specified graph, running the risk of identifying structures that are not present in the data [5]. More generally, as it can be shown that no single clustering algorithm will feature all desired statistical properties and perform well for all datasets, the field would benefit from additional methodologies [6].

One of the most widely used unsupervised clustering in general is k-means clustering, and it forms the basis of several methodologies, including scCCESS [7], SCCAF [8] and the single cell consensus clustering (SC3) algorithm [9]. To achieve robust and accurate results SC3 uses a consensus approach whereby a large number of parameter combinations are evaluated and subsequently combined. However, both the k-means clustering and the consensus algorithm come at significant computational costs: both the run time and memory use scale more than quadratically with the number of cells, prohibiting application to large datasets, which are becoming increasingly commonplace with ever improving sequencing technologies.

## Implementation

**Fig. 1:**
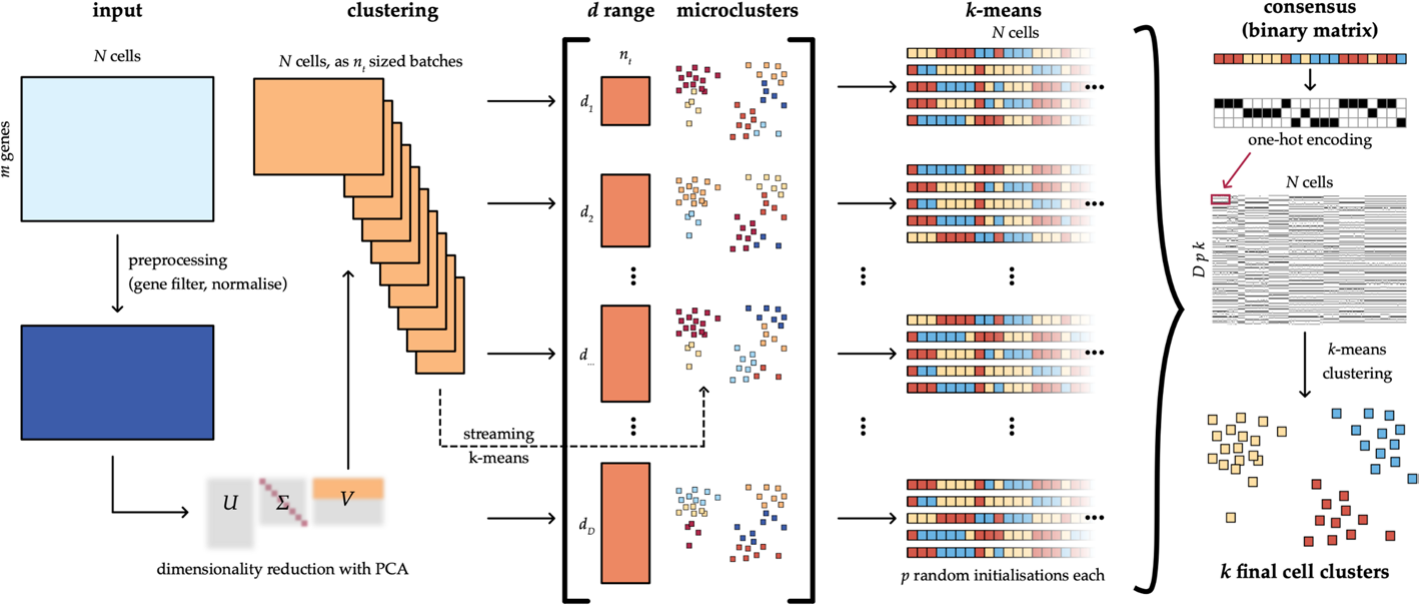
The SC3s framework for single cell consensus clustering. SC3s takes as input the gene-by-cell expression matrix, after preprocessing and dimensionality reduction via PCA using Scanpy commands. To achieve consensus clustering, SC3s attempts to combine the results of multiple clustering runs, where the number of principal components is changed (*d* range). All this information is then encoded into a binary matrix, which can be efficiently used to produce the final *k* cell clusters. The key difference from the original SC3 is that for each *d*, the cells are first grouped into microclusters which can be reused for multiple values of *k*, saving time in computation

Here, we present a new version of this algorithm, single cell consensus clustering with speed (SC3s), where several steps of the original workflow have been optimized to ensure that both run time and memory usage scale linearly with the number of cells (Fig. 1; Additional file 1: Fig. S1). This is achieved by using a streaming approach for the k-means clustering [10], as implemented in the *scikit-learn* package [11], which makes it possible to only process a small subset of cells in each iteration. Each of the subsets can be efficiently processed at constant time and memory. In addition, as part of an intermediary step, which was not part of the original method, a large number of microclusters are calculated. The microclusters can be reused for different choices of *k*, and this allows substantial savings when analyzing multiple values of *k*, something that is very common in practice during data exploration. We have also improved the consensus step by adopting a one-hot encoding approach [12], as opposed to the original co-association based method, on which the k-means clustering algorithm could be run more efficiently (Additional file 1: Fig. S2).

## Results

**Fig. 2:**
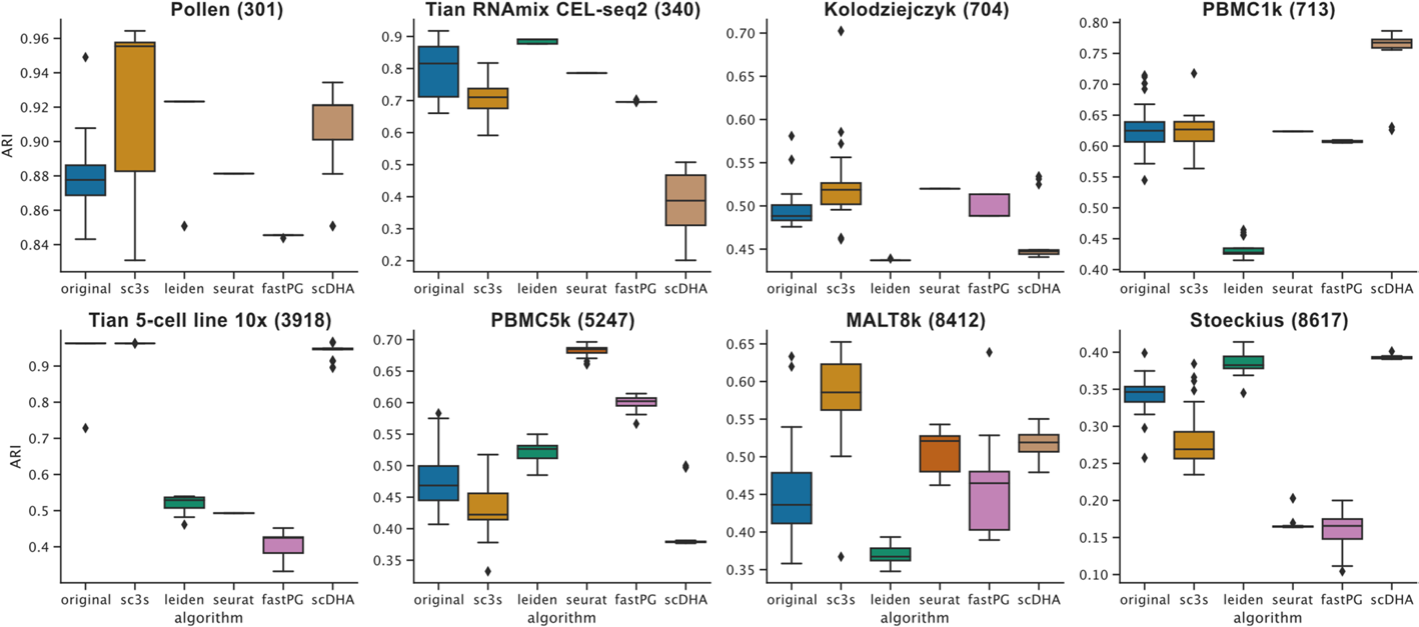
Clustering accuracy benchmarks on gold-standard datasets with < 10,000 cells. Boxplots show the ARI distribution across 25 realizations of each algorithm. Numbers in parentheses denote the cell count in the specified dataset. The performance of the original SC3 is shown in blue. Leiden refers to the algorithm of the same name as implemented in Scanpy. Seurat refers to its SNN modularity optimization clustering algorithm. ARI: Adjusted Rand index (ARI)

To evaluate the accuracy of SC3s we used eight datasets with < 10,000 cells where the cell labels are known or have been defined using orthogonal methods, allowing us to compare the results of the transcriptome clustering to a ground truth [9] (Additional file 1: Table S1). These benchmarks show that SC3s has an accuracy which is comparable to the original algorithm (Fig. 2), and that the performance is robust across a broad range of user-customisable parameters (Additional file 1: Figs. S3-S5). Finally, SC3s compares favorably against other clustering methodologies, such as Scanpy, Seurat, FastPG and scDHA, in terms of its accuracy, memory usage and runtime (Fig. 2; Additional file 1: Figs. S1, S6).

To examine the performance for large datasets, SC3s was benchmarked on the mouse organogenesis cell atlas dataset which contains 2,026,641 cells [1]. Processing, filtering and dimensionality reduction were performed as in the original publication, after which the clustering performance of SC3s was assessed. Compared to the other packages, SC3s was able to achieve both a short runtime and a low memory usage, whilst producing consistent clusters. For example, when compared to the Leiden algorithm, the peak memory usage was similar, but SC3s was ~ 18 times faster (20 min vs 6 h), even when evaluating five *k* values (Table 1). The slightly lower accuracy was expected because cell labels used for comparison originated from the Louvain algorithm, a method very similar to the Leiden algorithm, making them an imperfect ground truth. Visual inspection of the assigned labels also revealed that SC3s was able to capture the major structures identified by the authors (Additional file 1: Fig. S7).

**Table 1:** Runtime, memory and ARI performance benchmarked on the 2 million mouse organogenesis cell atlas dataset

Method	Runtime (hr:min:s)	Peak memory (GB)	ARI
SC3s (one k)	00:11:12	22.65	0.408
SC3s (one k)	00:21:01	26.45	0.400
Scanpy Leiden	05:27:17	33.83	0.536
SEURAT	01:53:54	91.36	0.257
FastPG	00:15:05	72.75	0.463

Results are the average of five iterations

## Conclusions

Overall, SC3s is a major improvement over its predecessor, and it represents a scalable and accurate alternative to the widely used neighborhood graph clustering methodologies. Moreover, it is integrated with the popular Scanpy package and utilizes the same underlying data structures [13], making it easy for users to incorporate into existing workflows and to make full use of upstream and downstream functionalities in the ecosystem. Thus, SC3s will allow researchers to analyze scRNAseq datasets as they scale to millions of cells.

### Availability and requirements

Project name: SC3s. Project home page: https://github.com/hemberg-lab/sc3s/. Operating system: Platform independent. Programming language: python. License: BSD-3. Other requirements: None. Restrictions to use by non-academics: None.

## Supplementary Information

**Additional file 1.** Contains **Fig S1-S7** which provides more details about SC3s performance, and **Table S1** which details the datasets used for benchmarking.

## Data Availability

All datasets used for benchmarking are available publically, and they are listed in Additional file 1: Table S1. The Python code for SC3s is licensed under a BSD-3 Clause License. Instructions to install from pip and conda channels are available on GitHub: https://github.com/hemberg-lab/sc3s.

## Abbreviations

scRNAseq: Single cell RNAseq
SC3: Single cell consensus clustering
SC3s: Single cell consensus clustering with speed
