# Supplementary material for: SC3s: efficient scaling of single cell consensus clustering to millions of cells
Source: BMC Bioinformatics. 2022 Dec 12;23:536. doi: 10.1186/s12859-022-05085-z (PMC9743492; doi:10.1186/s12859-022-05085-z)
Supplement: Supplementary file 1 — Additional file 1. Contains Fig S1-S7 which provides more details about SC3s performance, and Table S1 which details the datasets used for benchmarking. [file 12859_2022_5085_MOESM1_ESM.docx]

### Additional Files

**Figure S1: Runtime and memory performance relative to the number of cells.** For each dataset, five realizations of the SC3s (with one and five *k* values tested) and the other algorithms were plotted. The results for scDHA were omitted because they were at least twice the magnitude of the shown results (e.g. 1300 s and 2515 MB RAM for 5,247 cells).

**
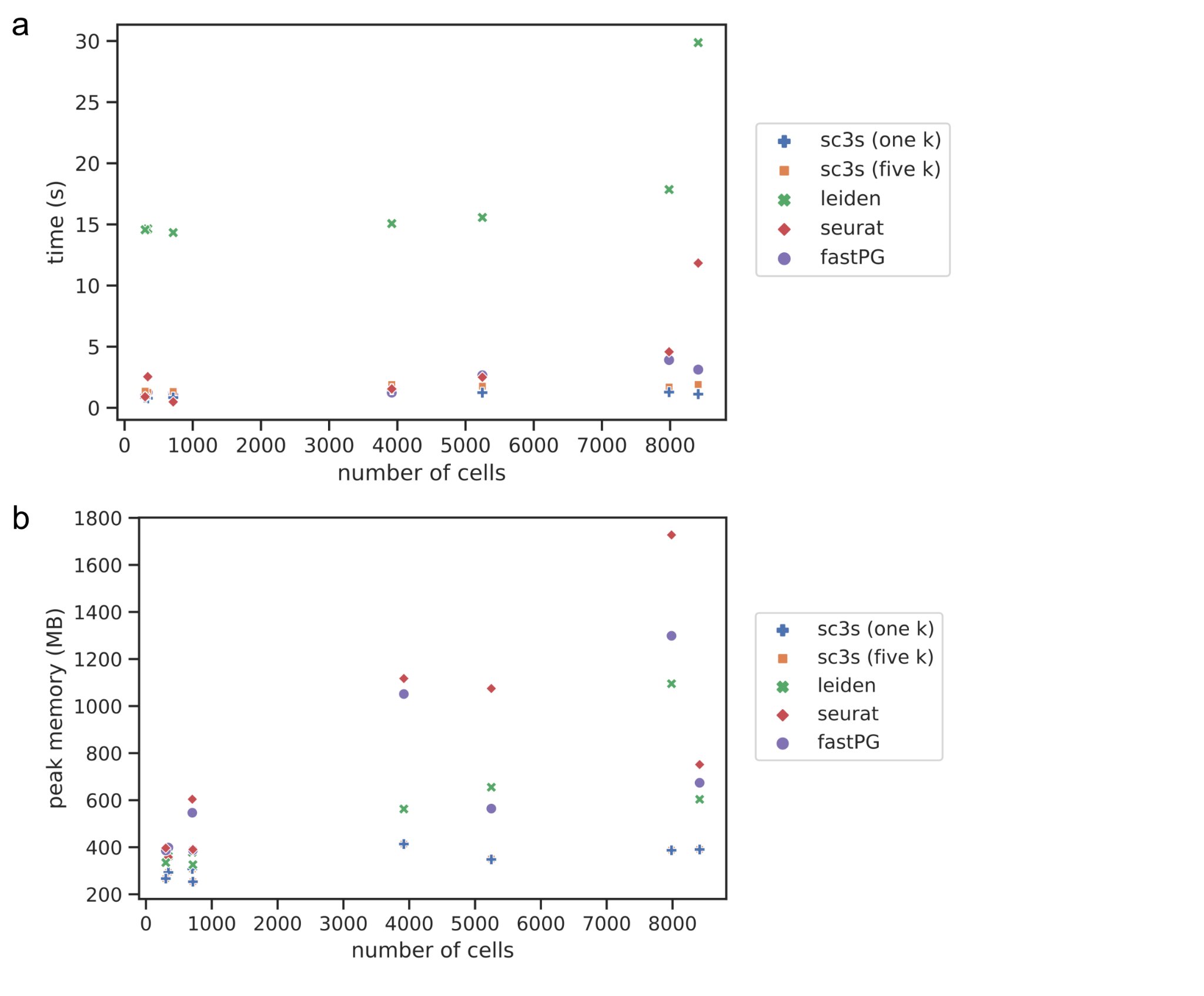
**

**Figure S2: Performance improvements of the one hot encoding approach for consensus clustering.** Runtime and memory usage is significantly reduced not only for the construction of the consensus matrix (a, b), but also its subsequent use to generate the final clusters (c, d).

**
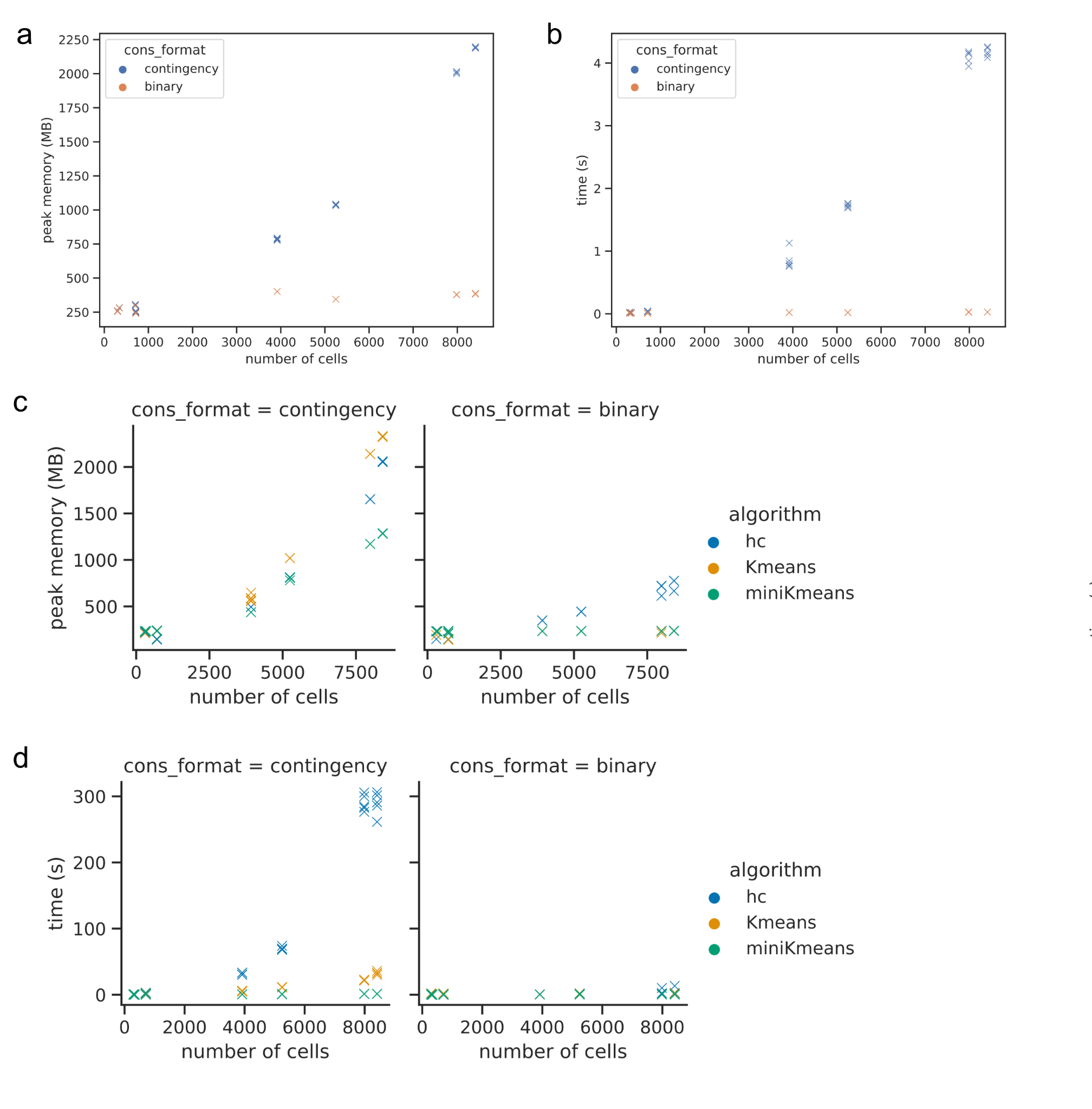
**

**Figure S3: Stability of SC3s clustering results across a wide range of d values (number of PCs).** The mean ARI performance with 95% confidence intervals is shown across 25 independent realizations for each *d* value. *d* values are plotted as a fraction of the true number of clusters k (panel a), and as a fraction of the number of cells (panel b). In general, a plateau of robust ARI performance is obtained once a sufficient number of PCs are included. The algorithm exhibits higher accuracy and more stability when more runs are used to obtain the consensus.

**
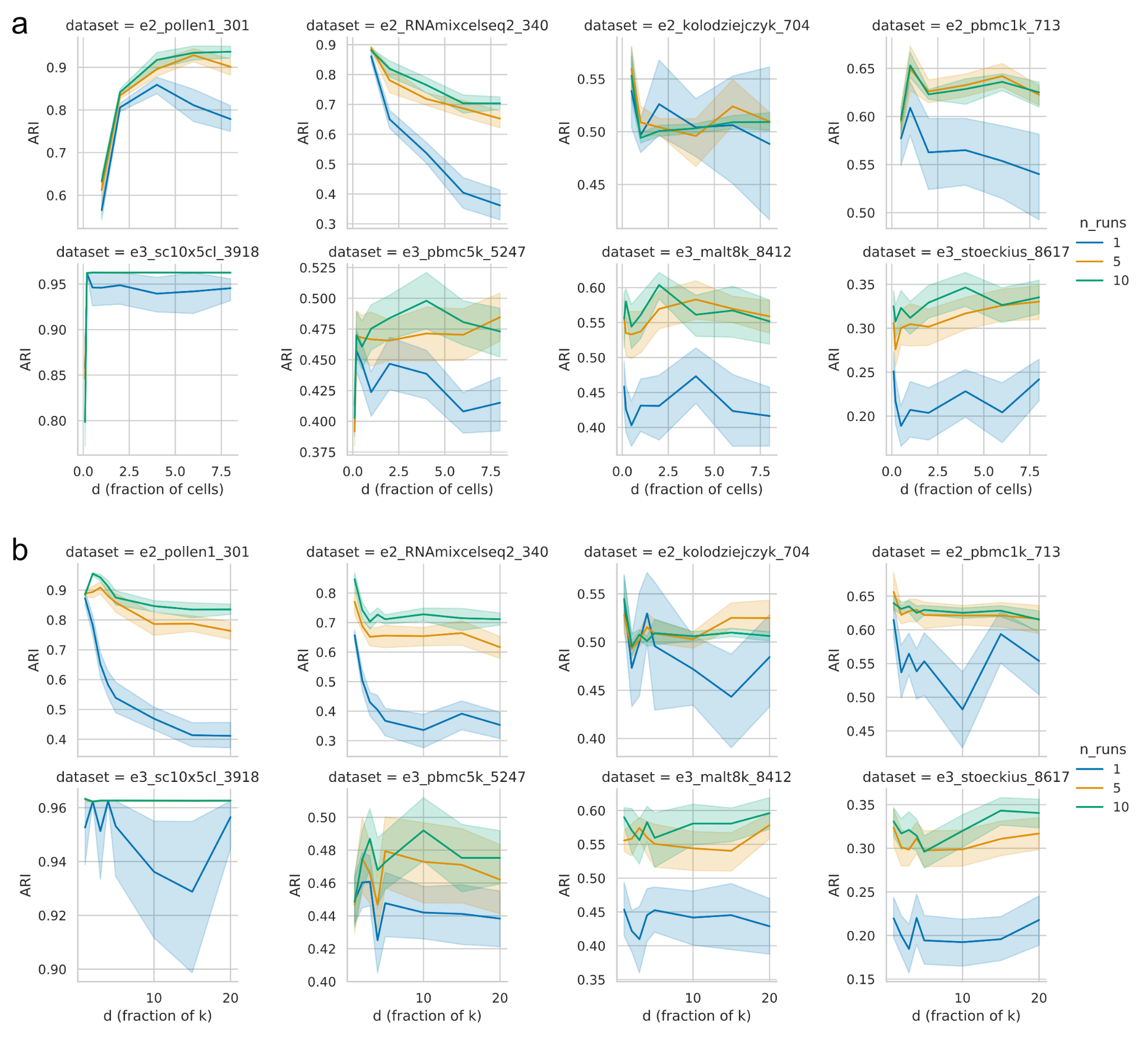
**

**Figure S4: Stability of SC3s clustering results with respect to the n_runs parameter.** *n_runs* denote the number of runs used to calculate the consensus. ARI performance of SC3s increases with a higher number of iterations run to obtain the consensus. 25 independent realizations were used for each dataset.


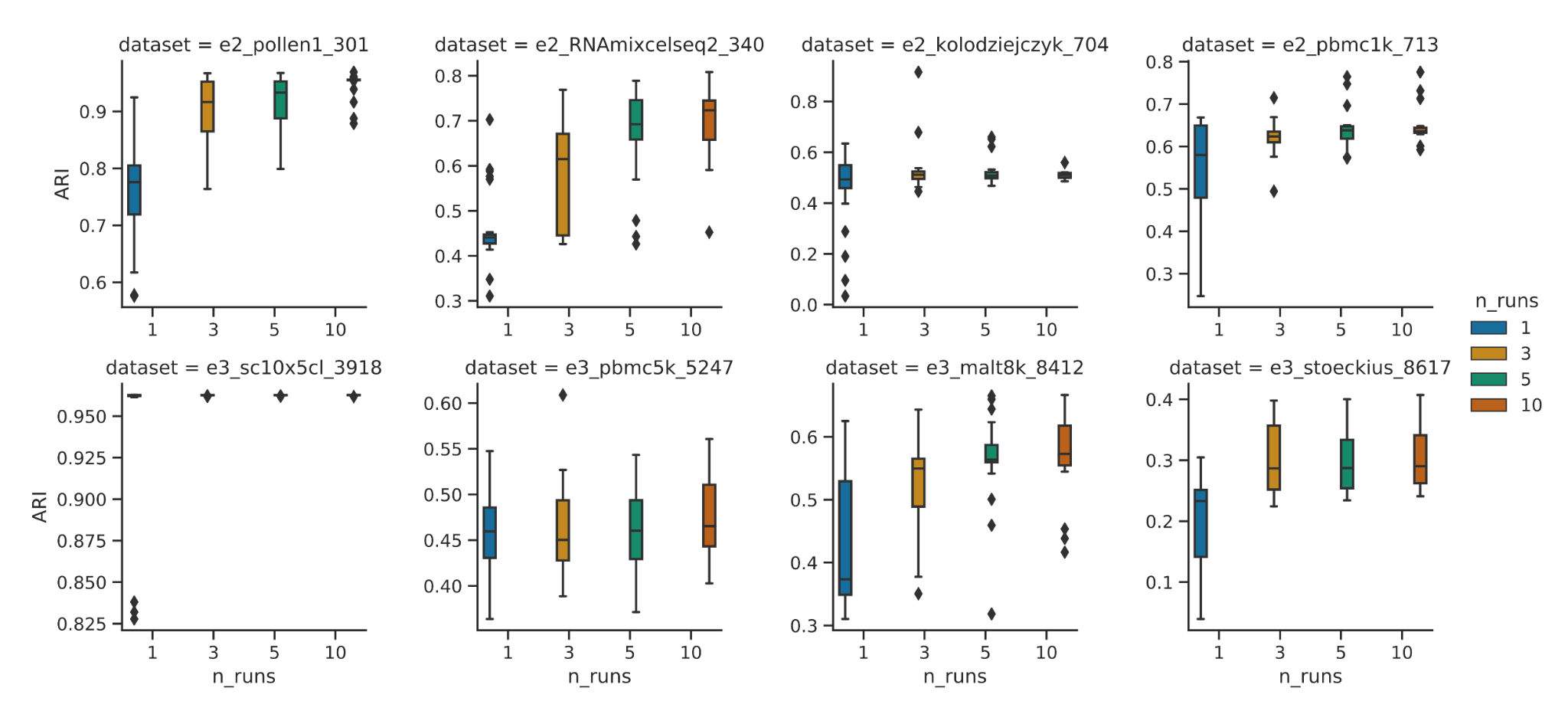


**Figure S5: Stability of SC3s clustering results with respect to the multiplier parameter.** The ARI performance is examined under a different number of microclusters, which was calculated as the true number of *k* multiplied by the multiplier value. 25 independent realizations were used for each dataset.

**
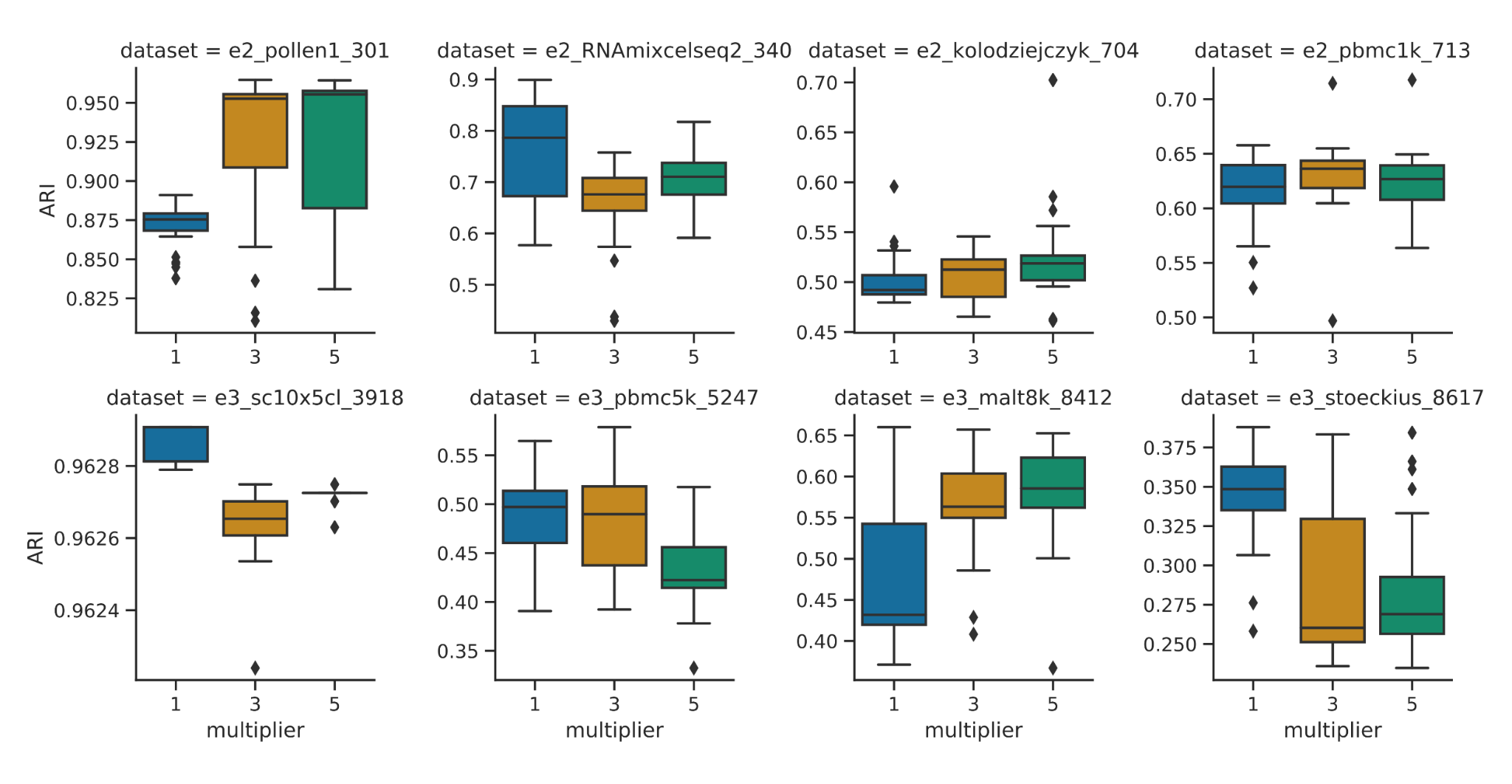
**

**Figure S6: ARI performance of Leiden algorithm, under different parameterisations.** Color denotes the number of neighbors used to construct the neighborhood graph, while the x-axis denotes the resolution parameter in the Leiden algorithm. 25 independent realizations each.


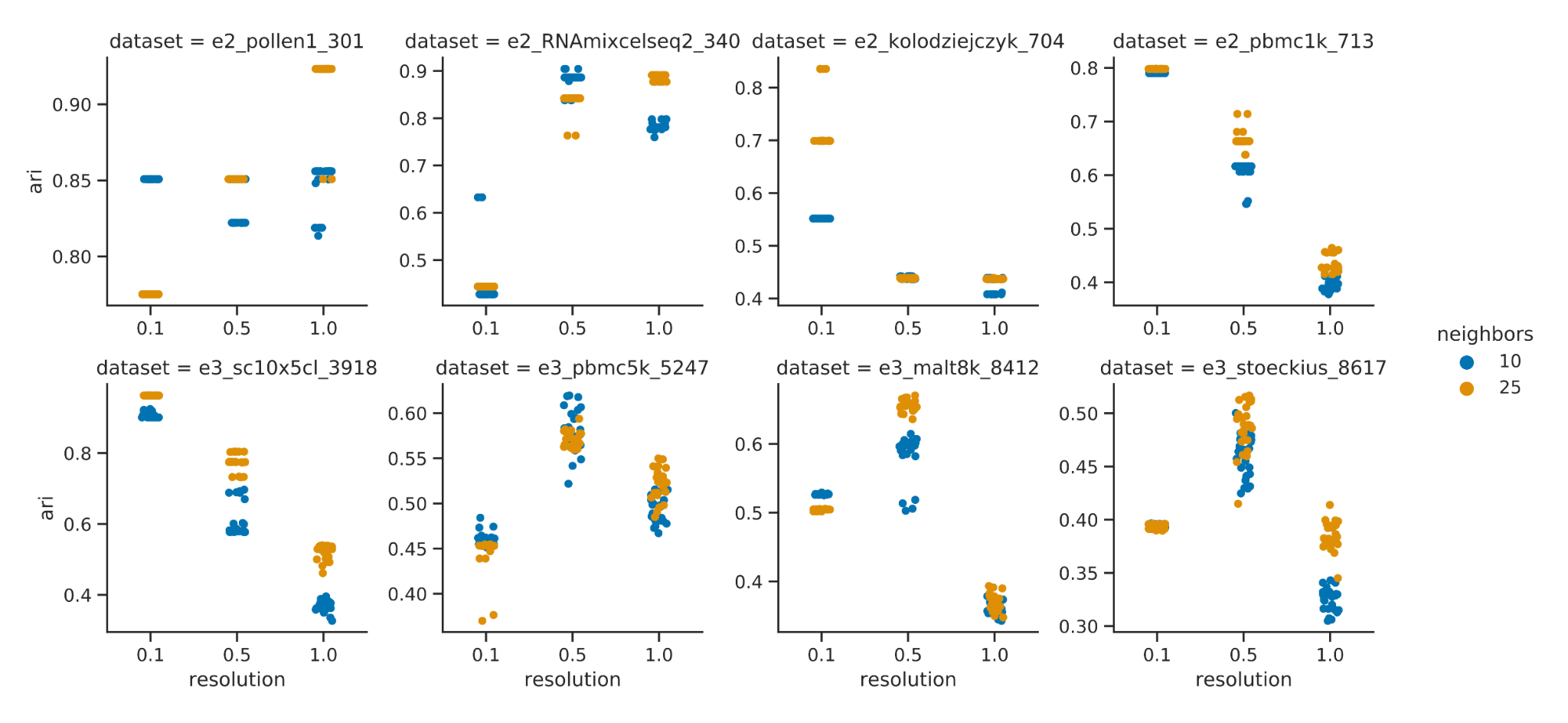


**Figure S7: Sankey diagram comparing the clusters reported by Cao et al (left) with clusters assigned by SC3s (right).** The width of the edges correspond to the number of cells common to the linked nodes. To focus only on the major structure in the data, only edges which contain above 20% of the cell types in one of the parent nodes were included.


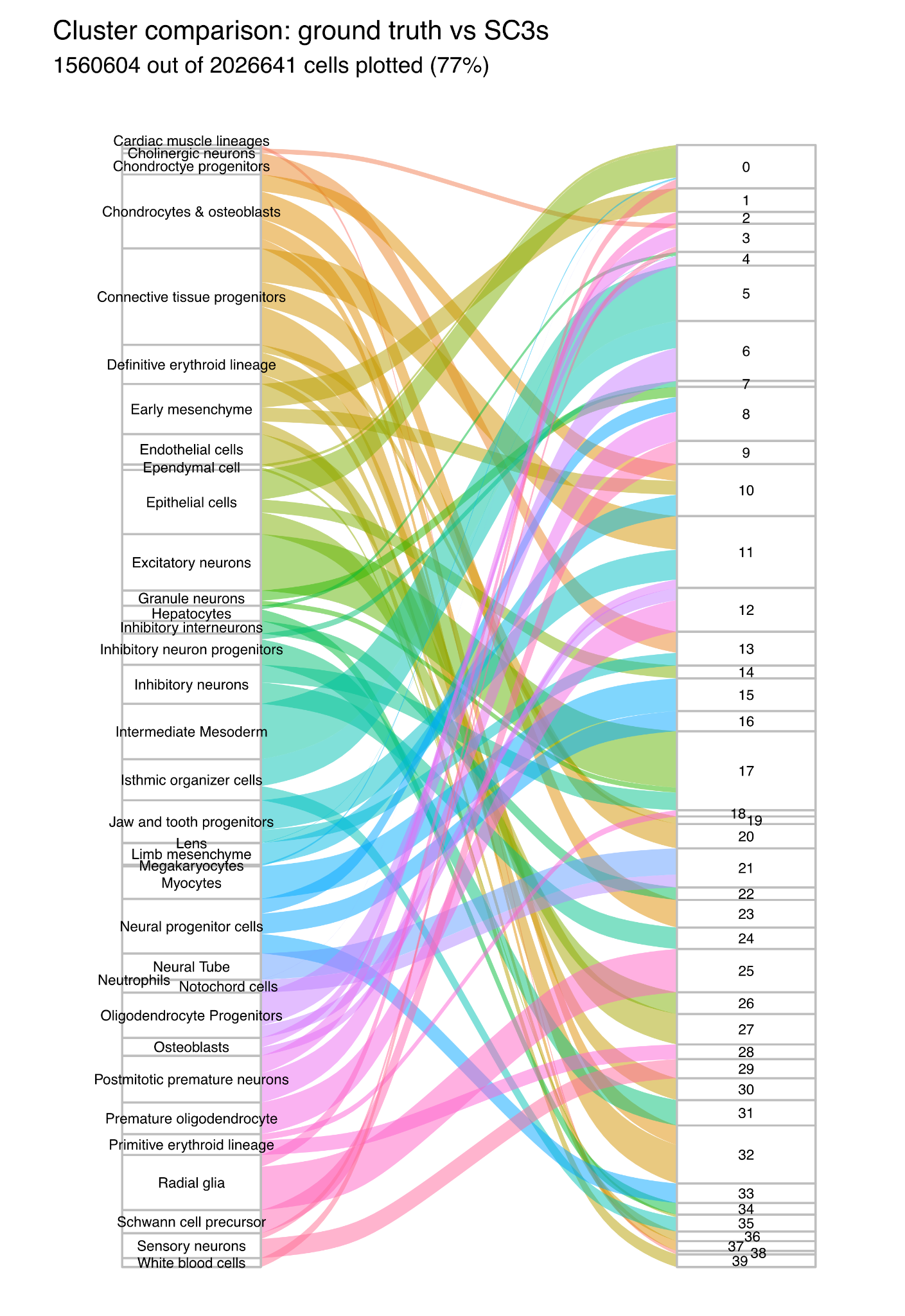


**Table S1: Datasets used for benchmarking**

| **Name** | **Identifier** | **Cell count** | **True k** | **Source** | **URL** |
| --- | --- | --- | --- | --- | --- |
| Pollen | e2_pollen_1 | 301 | 11 | Pollen A.A. *et al. ^1^* | https://hemberg-lab.github.io/scRNA.seq.datasets/human/tissues/#pollen |
| Tian RNAmix CEL-seq2 | e2_RNAmixcelseq2_340 | 340 | 7 | Tian, L. *et al.* *^2^* | https://github.com/LuyiTian/sc_mixology |
| Kolodziejczyk | e2_kolodziejczyk_704 | 704 | 3 | Kolodziejczyk, A. A. *et al.* *^3^* | https://hemberg-lab.github.io/scRNA.seq.datasets/mouse/esc/#kolodziejczyk |
| PBMC1k | e2_pbmc1k_713 | 713 | 6 | 10x Genomics | https://support.10xgenomics.com/single-cell-gene-expression/datasets/3.0.0/pbmc_1k_protein_v3 |
| Tian 5-cell line 10x | e3_sc10x5cl_3918 | 3918 | 5 | Tian, L. *et al.* *^2^* | https://github.com/LuyiTian/sc_mixology |
| PBMC5k | e3_pbmc5k_5247 | 5247 | 8 | 10x Genomics | https://support.10xgenomics.com/single-cell-gene-expression/datasets/3.0.2/5k_pbmc_protein_v3 |
| MALT8k | e3_malt8k_8412 | 8412 | 8 | 10x Genomics | https://support.10xgenomics.com/single-cell-gene-expression/datasets/3.0.0/malt_10k_protein_v3 |
| Stoeckius | e3_stoeckius_8617 | 8617 | 8 | Stoeckius, M. *et al. ^4^* | https://www.ncbi.nlm.nih.gov/geo/query/acc.cgi?acc=GSE100866 |
| MOCA | e6_moca_2mil | 2026641 | 38 | Cao, J. *et al. ^5^* | http://oncoscape.v3.sttrcancer.org/atlas.gs.washington.edu.mouse.rna |

1. Kolodziejczyk AA, Kim JK, Tsang JCH, Ilicic T, Henriksson J, Natarajan KN, et al. Single Cell RNA-Sequencing of Pluripotent States Unlocks Modular Transcriptional Variation. Cell Stem Cell. 2015;17:471–85.
2. Tian L, Dong X, Freytag S, Lê Cao K-A, Su S, JalalAbadi A, et al. Benchmarking single cell RNA-sequencing analysis pipelines using mixture control experiments. Nat Methods. 2019;16:479–87.
3. Pollen AA, Nowakowski TJ, Shuga J, Wang X, Leyrat AA, Lui JH, et al. Low-coverage single-cell mRNA sequencing reveals cellular heterogeneity and activated signaling pathways in developing cerebral cortex. Nat Biotechnol. 2014;32:1053–8.
4. Stoeckius M, Hafemeister C, Stephenson W, Houck-Loomis B, Chattopadhyay PK, Swerdlow H, et al. Simultaneous epitope and transcriptome measurement in single cells. Nat Methods. 2017;14:865–8.
5. Cao J, Spielmann M, Qiu X, Huang X, Ibrahim DM, Hill AJ, et al. The single-cell transcriptional landscape of mammalian organogenesis. Nature. 2019;566:496–502.
